# MORTALITY RISK AMONG OLDER VETERANS AND NONVETERANS: THE IMPORTANCE OF COMBAT STATUS

**DOI:** 10.1093/geroni/igac059.597

**Published:** 2022-12-20

**Authors:** Scott Landes, Jennifer Piazza

**Affiliations:** Syracuse University, Syracuse, New York, United States; California State University, Fullerton, Fullerton, California, United States

## Abstract

Over 17.4 million Americans have served in the U.S. armed forces. Although long-term mortality risk is reported to be higher among older veterans than nonveterans, research does differentiate whether there is variation by combat status. This study examined later-life mortality rates among nonveterans, noncombat veterans, and combat veterans. Data were from Wave 2 of the Midlife Development in the United States Survey (N = 4,633). Participants included 3832 nonveterans, 584 noncombat veterans, and 217 combat veterans. Mortality rates did not differ when comparing nonveterans and noncombat veterans. Combat veterans, however, had a higher risk of mortality than did than nonveterans. Combat experience is a determinant of long-term mortality risk among veterans. Future studies should account for combat status when comparing health and mortality between veterans and nonveterans. Because of their heightened mortality risk, combat veterans should be provided with additional services during and after their time in the armed forces.

